# Durable treatment-free remission in patients with chronic myeloid leukemia in chronic phase following frontline nilotinib: 96-week update of the ENESTfreedom study

**DOI:** 10.1007/s00432-018-2604-x

**Published:** 2018-02-22

**Authors:** David M. Ross, Tamas Masszi, María Teresa Gómez Casares, Andrzej Hellmann, Jesper Stentoft, Eibhlin Conneally, Valentin Garcia-Gutierrez, Norbert Gattermann, Philipp D. le Coutre, Bruno Martino, Susanne Saussele, Francis J. Giles, Jerald P. Radich, Giuseppe Saglio, Weiping Deng, Nancy Krunic, Véronique Bédoucha, Prashanth Gopalakrishna, Andreas Hochhaus

**Affiliations:** 1 Division of Haematology, SA Pathology, Room 6E359, Royal Adelaide Hospital, 1 Port Rd, Adelaide, SA 5000 Australia; 2 Semmelweis University, Budapest, Hungary; 3 Hospital Universitario de Gran Canaria Dr Negrín, Las Palmas de Gran Canaria, Spain; 4 Medical University of Gdańsk, Gdańsk, Poland; 5 Aarhus University Hospital, Aarhus, Denmark; 6 St James’s Hospital, Dublin, Ireland; 7 Hospital Universitario Ramón y Cajal, IRYCIS, Madrid, Spain; 8 Universitätsklinikum Düsseldorf, Düsseldorf, Germany; 9 Charité-Universitätsmedizin Berlin, Berlin, Germany; 10 Azienda Ospedaliera Bianchi Melacrino Morelli, Reggio Calabria, Italy; 11 III. Med. Klinik, Medizinische Fakultät Mannheim der Universität Heidelberg, Mannheim, Germany; 12 Developmental Therapeutics Consortium, Chicago, IL USA; 13 Clinical Research Division, Fred Hutchinson Cancer Research Center, Seattle, WA USA; 14 University of Turin, Orbassano, Italy; 15 Novartis Pharmaceuticals Corporation, East Hanover, NJ USA; 16 Novartis Institutes for BioMedical Research, Cambridge, MA USA; 17 Novartis Pharma AG, Basel, Switzerland; 18 Abteilung Hämatologie/Onkologie, Universitätsklinikum Jena, Jena, Germany

**Keywords:** Chronic myeloid leukemia, Treatment-free remission, Predictors of TFR, Nilotinib, Frontline, Clinical trial

## Abstract

**Purpose:**

ENESTfreedom is evaluating treatment-free remission (TFR) following frontline nilotinib in patients with chronic myeloid leukemia (CML) in chronic phase. Following our primary analysis at 48 weeks, we here provide an updated 96-week analysis.

**Methods:**

Attempting TFR required ≥ 3 years of nilotinib, a molecular response of MR^4.5^ [*BCR-ABL1* ≤ 0.0032% on the International Scale (*BCR-ABL1*^IS^)], and sustained deep molecular response (DMR) during a 1-year consolidation phase. Patients restarted nilotinib following loss of major molecular response (MMR; *BCR-ABL1*^IS^ ≤ 0.1%).

**Results:**

Ninety-six weeks after stopping treatment (3.6-year median prior nilotinib duration), 93 of 190 patients (48.9%) remained in TFR. Of 88 patients who restarted nilotinib following loss of MMR, 87 regained MMR and 81 regained MR^4.5^ by the data cut-off. Ninety-six-week TFR rates were 61.3, 50.0, and 28.6% in patients with low, intermediate, and high Sokal risk scores at diagnosis, respectively. Patients consistently in MR^4.5^ during consolidation had higher TFR rates (50.6%) than patients with ≥ 1 assessment without MR^4.5^ during consolidation (35.0%). In a landmark analysis, 96-week TFR rates for patients with MR^4.5^, MR^4^ (*BCR-ABL1*^IS^ ≤ 0.01%) but not MR^4.5^, and MMR but not MR^4^ at TFR week 12 were 82.6, 23.1, and 0%, respectively. There were no reports of disease progression or death due to CML; overall adverse event frequency decreased following TFR. Within the follow-up period, TFR did not adversely affect disease outcomes.

**Conclusions:**

These results demonstrate the feasibility and durability of TFR following frontline nilotinib and emphasize the importance of sustained DMR for TFR.

## Introduction

For patients with chronic myeloid leukemia in chronic phase (CML-CP) who have achieved a stable deep molecular response (DMR) using BCR-ABL1 tyrosine kinase inhibitors (TKIs), treatment-free remission (TFR) following TKI cessation is an emerging goal (Hughes and Ross 2016; Mahon et al. 2010; Etienne et al. 2017; Rousselot et al. 2014; Ross et al. 2013; Lee et al. 2016; Mori et al. 2015; Hochhaus et al. 2017a; Hughes et al. 2016; Imagawa et al. 2015; Nakamae et al. 2017; Saussele et al. 2017; Rea et al. 2017; Boquimpani et al. 2014; Villemagne Sanchez et al. 2018). Potential reasons why patients or physicians may wish to attempt TFR include the possibility of reducing adverse events (AEs), avoiding long-term toxicities, reducing costs, improving quality of life, the convenience of having fewer medications to take, and the ability to plan pregnancies (Boquimpani et al. 2014; Villemagne Sanchez et al. 2018; Jiang et al. 2016). In the original Stop Imatinib (STIM1) trial, which has the longest TFR follow-up to date, patients attempted TFR following achievement of sustained undetectable minimal residual disease with imatinib (Etienne et al. 2017; Mahon et al. 2010). Sixty months after TKI cessation, the Kaplan–Meier-estimated molecular recurrence-free survival rate was 38%, and most instances of molecular recurrence occurred during the first 6 months. Other TFR studies have reported comparable results (Ross et al. 2013; Rousselot et al. 2014; Lee et al. 2016; Mori et al. 2015; Hochhaus et al. 2017a; Imagawa et al. 2015; Nakamae et al. 2017; Saussele et al. 2017; Rea et al. 2017; Hughes et al. 2016). In light of the success of TFR studies and the possible advantages that TFR might confer, the US National Comprehensive Cancer Network and the European Society for Medical Oncology have published guidelines that include criteria for attempting TFR outside of clinical trials (National Comprehensive Cancer Network 2017; Hochhaus et al. 2017b).

For patients with newly diagnosed CML-CP, frontline treatment with the second-generation TKI nilotinib can lead to a higher rate of stable DMR (and TFR eligibility) than frontline treatment with imatinib (Hochhaus et al. 2015). In the Evaluating Nilotinib Efficacy and Safety in Clinical Trials—Newly Diagnosed Patients (ENESTnd) study, nilotinib resulted in higher 5-year cumulative rates of MR^4.5^ [*BCR-ABL1* ≤ 0.0032% on the International Scale (*BCR-ABL1*^IS^)] than imatinib (nilotinib 300 mg twice daily, 54%; nilotinib 400 mg twice daily, 52%; imatinib, 31%) (Hochhaus et al. 2016), and an estimated ≥ 80% of patients achieving MR^4.5^ with nilotinib maintained the response for ≥ 1 year (Hochhaus et al. 2015). The ENESTfreedom study is the first study specifically to assess the feasibility of TFR in patients with CML-CP achieving sustained DMR with frontline nilotinib (Hochhaus et al. 2017a). The primary analysis reported that 48 weeks after attempting TFR, 51.6% of patients remained off treatment without loss of major molecular response (MMR; *BCR-ABL1*^IS^ ≤ 0.1%). This is comparable to TFR rates following imatinib treatment, despite a shorter duration of prior TKI therapy (median of 3.6 years in ENESTfreedom vs ≈ 5–8 years in prior imatinib studies) (Hochhaus et al. 2017a; Etienne et al. 2017; Ross et al. 2013; Rousselot et al. 2014; Lee et al. 2016; Mori et al. 2015). Based on the results of the primary analysis from ENESTfreedom, as well as results from the ENESTop study of TFR following second-line nilotinib, nilotinib became the first TKI with TFR in its product label (Hochhaus et al. 2017a; Mahon et al. 2018; Novartis Pharmaceuticals Corporation 2017a, b). We now report an updated analysis of the ENESTfreedom study based on 96 weeks of follow-up in the TFR phase.

## Methods

### Study design

**Fig. 1:**
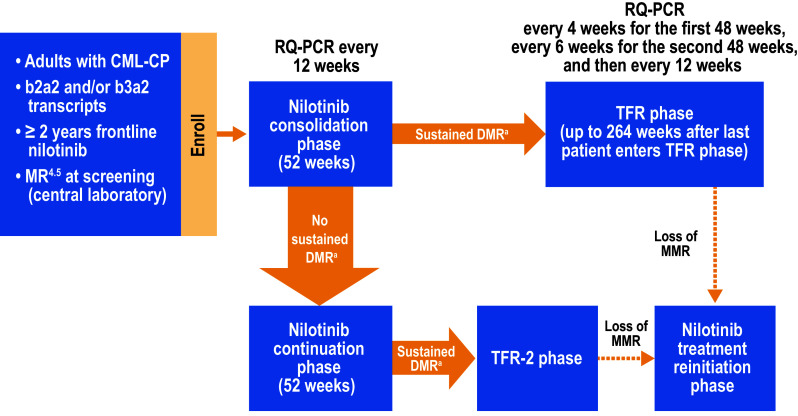
ENESTfreedom study design. *CML-CP* chronic myeloid leukemia in chronic phase, *DMR* deep molecular response, *MMR* major molecular response [*BCR-ABL1* on the International Scale (*BCR-ABL1*^IS^) ≤ 0.1%], *MR*^*4*^ *BCR-ABL1*^IS^ ≤ 0.01%, *MR*^*4.5*^ *BCR-ABL1*^IS^ ≤ 0.0032%, *RQ-PCR* real-time quantitative polymerase chain reaction, *TFR* treatment-free remission. ^a^Sustained DMR was defined as the following (in the last 4 quarterly RQ-PCR assessments): MR^4.5^ in the last assessment, ≤ 2 assessments between MR^4^ and MR^4.5^, and no assessment worse than MR^4^

ENESTfreedom is an ongoing, single-arm, phase 2 study (NCT01784068) that has been previously described (Hochhaus et al. 2017a). Briefly, adult patients (aged ≥ 18 years) with Philadelphia chromosome–positive CML-CP, ≥ 2 years of frontline nilotinib therapy, and MR^4.5^ were eligible to enroll. Patients having received prior interferon alfa therapy or > 4 weeks of any other BCR-ABL1 TKI were not eligible. Following enrollment, patients entered a 1-year consolidation phase (Fig. 1) during which they continued nilotinib treatment and were monitored by real-time quantitative polymerase chain reaction (RQ-PCR) every 12 weeks. Patients who maintained DMR during the consolidation phase, meaning no assessment worse than MR^4^ (*BCR-ABL1*^IS^ ≤ 0.01%), ≤ 2 assessments between MR^4^ and MR^4.5^, and MR^4.5^ in the last assessment, could enter the TFR phase. Patients in the TFR phase were monitored by RQ-PCR every 4 weeks during the first 48 weeks, every 6 weeks during the next 48 weeks, and every 12 weeks thereafter up to 264 weeks after the last patient entered the TFR phase. Nilotinib reinitiation was triggered by any single assessment showing *BCR-ABL1*^IS^ > 0.1% (loss of MMR). Patients in the reinitiation phase were monitored every 4 weeks for the first 24 weeks and every 12 weeks thereafter (or as clinically indicated for patients who had not regained MMR) to assess molecular response.

### Updated and additional analyses

Here, we report updated results based on a data cut-off date of 31 October 2016, at which time all patients who entered the TFR phase had completed 96 weeks of TFR, transitioned to the reinitiation phase, or discontinued from the study. The TFR rate was calculated as a percentage with an exact 95% Clopper–Pearson confidence interval. Treatment-free survival (TFS) was defined as the time from TFR start to the loss of MMR, nilotinib reinitiation for any reason, progression to accelerated phase/blast crisis (AP/BC), or death from any cause and was estimated using the Kaplan–Meier method. For patients without any such event, TFS was censored at the date of last assessment. Molecular response rates in patients who entered retreatment due to loss of MMR in the TFR phase were calculated as cumulative incidences.

To investigate factors potentially associated with TFR, we stratified all patients attempting TFR by Sokal risk score at diagnosis and by MR^4.5^ stability during the consolidation phase and calculated TFR rates at 48 and 96 weeks in each subgroup. Patients’ Sokal risk scores at diagnosis were not collected at study entry but rather retrospectively following a protocol amendment; therefore, data on Sokal risk at diagnosis were not available for all patients. MR^4.5^ stability during the consolidation phase was defined based on patients’ responses during the 4 protocol-mandated RQ-PCR assessments collected during that phase. TFR rates were evaluated in patients with MR^4.5^ in all consolidation phase assessments vs those with ≥ 1 assessment worse than MR^4.5^. To assess the association between *BCR-ABL1*^IS^ levels at week 12 of the TFR phase and future TFR maintenance, we stratified patients remaining in the TFR phase for ≥ 12 weeks by *BCR-ABL1*^IS^ level at week 12 (MR^4.5^, MR^4^ but not MR^4.5^, or MMR but not MR^4^) and calculated TFR rates at 48 and 96 weeks. Patients with missing *BCR-ABL1*^IS^ values at week 12 were excluded from this analysis.

Updated safety data included a characterization of AEs across the consolidation phase and the first and second 48 weeks of the TFR phase for patients remaining in the TFR phase for > 48 weeks. The musculoskeletal-pain AE grouping included events reported using the preferred terms of myalgia, arthralgia, bone pain, spinal pain, pain in extremity, and musculoskeletal pain. Time to first musculoskeletal-pain event among patients entering the TFR phase was estimated using the Kaplan–Meier method. AEs were assessed according to the Common Terminology Criteria for Adverse Events version 4.03.

### Ethical approval

This study was designed and conducted in accordance with the ethical principles of the Declaration of Helsinki and the ICH Harmonized Tripartite Guidelines for Good Clinical Practice. An independent ethics committee or institutional review board for each study center reviewed the study protocol and its amendments. All patients provided written informed consent before any study procedures and in accordance with local laws and regulations.

## Results

### Patients

**Fig. 2:**
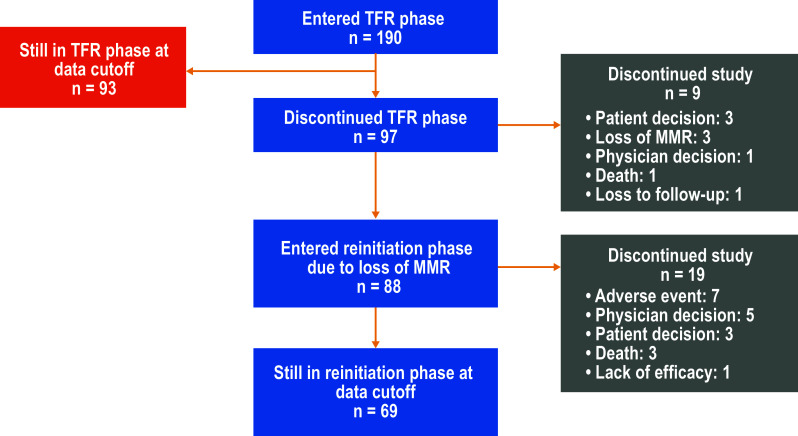
Patient flow and disposition by the 96-week data cut-off date. *MMR* major molecular response (*BCR-ABL1* ≤ 0.1% on the International Scale), *TFR* treatment-free remission

Patients who entered the TFR phase (*n* = 190) had a median age at study entry of 55 years (range 21–86 years), median total nilotinib duration prior to entering the TFR phase of 43.5 months (range 32.9–88.7 months), and a median time from first MR^4.5^ until entering the TFR phase of 30.4 months (range 12.3–83.0 months). The Sokal score at diagnosis was low in 62 patients (32.6%), intermediate in 50 patients (26.3%), high in 28 patients (14.7%), and unavailable for the remaining patients. The median duration of follow-up in the TFR phase at the 96-week data cut-off date was 75.9 weeks (range 8.4–133.0 weeks). At the data cut-off date, of the 190 patients who entered the TFR phase, 93 (48.9%) remained in the TFR phase, 88 (46.3%) reinitiated nilotinib, and 9 (4.7%) discontinued from the study while in the TFR phase (Fig. 2). Of the 88 patients who entered the reinitiation phase by the data cut-off, 69 (78.4%) remained in this phase and 19 (21.6%) had discontinued from the study.

### Treatment-free remission and response to retreatment

**Fig. 3:**
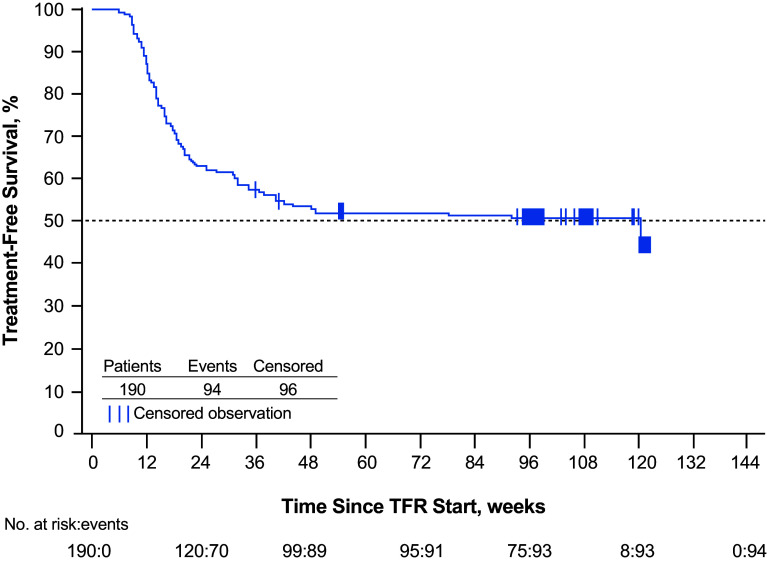
Kaplan–Meier-estimated TFS among all patients who entered the TFR phase. ^a^*MMR* major molecular response (*BCR-ABL1* ≤ 0.1% on the International Scale), *TFR* treatment-free remission, *TFS* treatment-free survival. ^a^TFS was defined as the time from the start of TFR until the earliest of any of the following: loss of MMR, reinitiation of nilotinib for any reason, progression to accelerated phase/blast crisis, or death due to any cause. By the data cut-off date, one patient had lost MMR at week 120, at which time only eight patients were considered at risk, resulting in the artificial drop seen at the end of the curve

**Fig. 4:**
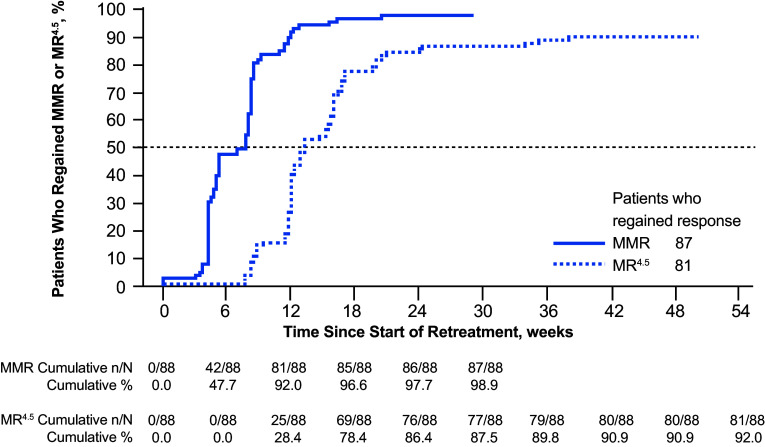
Cumulative incidence of MMR and MR^4.5^ regained after nilotinib reinitiation. ^a^*MMR* major molecular response [*BCR-ABL1* ≤ 0.1% on the International Scale (*BCR-ABL1*^IS^)], *MR*^*4.5*^ *BCR-ABL1*^IS^ ≤ 0.0032%. ^a^Of the 88 patients who reinitiated treatment, 1 discontinued from the study due to patient decision without regaining MMR 7.1 weeks after reinitiating treatment and the remaining 87 regained MMR on nilotinib

At week 96 of the TFR phase, 93 of 190 patients [48.9% (95% CI 41.6–56.3%)] remained off treatment and in MMR; 88 were also in MR^4.5^. A total of five patients who were in the TFR phase at 48 weeks were no longer in this phase at 96 weeks; 3 of these patients lost MMR after 48 weeks (at 54, 78, and 92 weeks), while the remaining 2 discontinued from the study without MMR loss (1 each due to patient decision and loss to follow-up). In addition to the three patients no longer in MMR at 96 weeks, a fourth lost MMR at 120 weeks; all four of these patients had lost MR^4.5^ and MR^4^ in the first 48 weeks of TFR. The estimated TFS rate at 96 weeks was 50.9% (95% CI 43.6–57.8%), and the Kaplan–Meier-estimated median TFS was 120 weeks (95% CI 36.9 weeks—not estimable; Fig. 3). Of the 88 patients who restarted nilotinib therapy due to MMR loss, 87 (98.9%) regained MMR and 81 (92.0%) also regained MR^4.5^ by the data cut-off date; the median time to regain MMR and MR^4.5^ was 7.0 and 13.1 weeks, respectively (Fig. 4). As previously described (Hochhaus et al. 2017a), the single patient who did not regain MMR discontinued from the study due to patient decision 7 weeks after reinitiating treatment. Of the 6 patients who regained MMR but not MR^4.5^, 1 remained on study and 5 discontinued from the study by the data cut-off date, between 5 and 25 weeks after nilotinib reinitiation (2 due to AEs, 1 due to lack of efficacy, and 2 due to individual decisions).

### Analysis of factors potentially associated with TFR

At both week 48 and 96 of the TFR phase, patients with low Sokal risk scores at diagnosis had higher TFR rates (62.9 and 61.3%, respectively) than those with intermediate (50.0 and 50.0%) or high (32.1 and 28.6%) scores at diagnosis (Table 1). Of the 190 patients who entered the TFR phase, 170 had MR^4.5^ in all four assessments during the consolidation phase, while the remaining 20 had ≥ 1 assessment worse than MR^4.5^. The TFR rate in the patients with MR^4.5^ in all assessments vs those lacking MR^4.5^ in ≥ 1 assessment was 52.9 vs 40.0% at 48 weeks and 50.6 vs 35.0% at 96 weeks, respectively.

**Table 1:** TFR rates in patient subgroups

TFR rate, *n*/*N* (% [95% CI])	TFR population (*n* = 190)	
	48 weeks	96 weeks
Sokal risk score at diagnosis		
Low	39/62 (62.9 [49.7–74.8])	38/62 (61.3 [48.1–73.4])
Intermediate	25/50 (50.0 [35.5–64.5])	25/50 (50.0 [35.5–64.5])
High	9/28 (32.1 [15.9–52.4])	8/28 (28.6 [13.2–48.7])
Unknown	25/50 (50.0 [35.5–64.5])	22/50 (44.0 [30.0-58.7])
*BCR-ABL1*^IS^ level in the consolidation phase		
MR^4.5^ in all assessments	90/170 (52.9 [45.2–60.6])	86/170 (50.6 [42.8–58.3])
≥ 1 assessment of MR^4^ but not MR^4.5^	8/20 (40.0 [19.1–63.9])	7/20 (35.0 [15.4–59.2])

*MR*^*4*^ *BCR-ABL1* on the International Scale (*BCR-ABL1*^IS^) ≤ 0.01%, *MR*^*4.5*^ *BCR-ABL1*^IS^ ≤ 0.0032%, *TFR* treatment-free remission

**Fig. 5:**
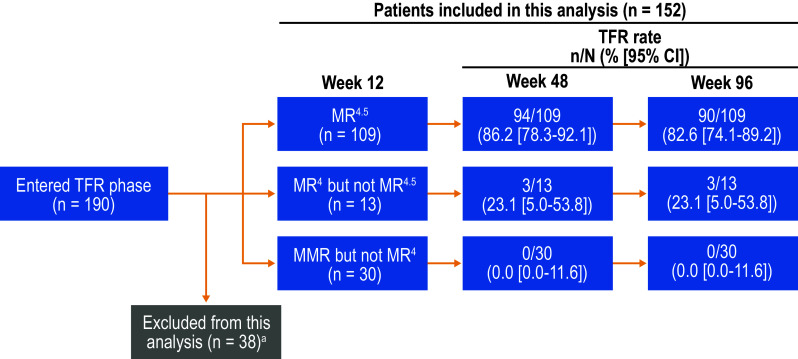
TFR rates according to response level at TFR week 12. *MMR* major molecular response [*BCR-ABL1* on the International Scale (*BCR-ABL1*^IS^) ≤ 0.1%], *MR*^*4*^ *BCR-ABL1*^IS^ ≤ 0.01%, *MR*^*4.5*^ *BCR-ABL1*^IS^ ≤ 0.0032%, *TFR* treatment-free remission. ^a^Patients who lost MMR, reinitiated nilotinib, or discontinued from the TFR phase by week 12 and patients without available *BCR-ABL1* values at week 12 were excluded from this analysis

There were 152 patients who remained in the TFR phase for ≥ 12 weeks and had evaluable *BCR-ABL1*^IS^ values at week 12. Of these patients, 109 had MR^4.5^ at week 12 of the TFR phase, 13 had MR^4^ but not MR^4.5^, and 30 had MMR but not MR^4^ (Fig. 5). The TFR rates in patients with MR^4.5^, MR^4^ but not MR^4.5^, and MMR but not MR^4^ at 12 weeks were 86.2, 23.1, and 0% at 48 weeks and 82.6, 23.1, and 0% at 96 weeks, respectively. Among patients with MMR but not MR^4^ at 12 weeks, the last patient to lose MMR did so at 48 weeks.

### Safety

Eight deaths were reported on study by the data cut-off date, three of which occurred after the 48-week data cutoff (Table 2): one death from respiratory failure during the treatment reinitiation phase and two deaths from non-CML cancers > 30 days after study discontinuation. To date, no deaths due to CML or progressions to AP/BC have been reported in the TFR population.

**Table 2:** Deaths reported during each study phase^a^

Deaths, *n* (%)	Consolidation phase (*N* = 215)	TFR phase (*n* = 190)	Reinitiation phase (*n* = 88)	Posttreatment follow-up^b^
Total	2 (0.9)	1 (0.5)	3 (3.4)	2
Cardiac arrest	1 (0.5)	0	0	0
Suicide	1 (0.5)	0	0	0
Acute myocardial infarction	0	0	1 (1.1)	0
Respiratory failure	0	0	1 (1.1)^c^	0
Other cancers	0	0	0	2^b,c^
Unknown cause	0	1 (0.5)	1 (1.1)	0

*TFR* treatment-free remission

^a^Median duration by the 96-week data cut-off date was 52.1 weeks in the consolidation phase, 75.9 weeks in the TFR phase, and 85.0 weeks in the reinitiation phase

^b^Deaths were reported > 30 days after patients discontinued from the study

^c^New deaths reported since the 48-week data cut-off date

Among patients who remained in the TFR phase for > 48 weeks (*n* = 100), the overall frequency of AEs decreased from 85.0% during the consolidation phase to 76.0% during the first 48 weeks of the TFR phase and 62.0% during the second 48 weeks of the TFR phase. In this group of patients, the most frequent all-grade AEs reported during the second 48 weeks of TFR were nasopharyngitis (9.0%) and back pain (5.0%). Overall, among these 100 patients, cardiovascular events were reported in 3 during the consolidation phase, 2 during the first 48 weeks of TFR, and 1 during the second 48 weeks of TFR (Table 3); the frequency of AEs in the musculoskeletal-pain grouping increased upon starting TFR (34.0% during the first 48 weeks of TFR vs 17.0% during the consolidation phase) but then decreased as TFR continued (9.0% during the second 48 weeks of TFR). At the time of the 96-week data cut-off, with < 50% of patients experiencing musculoskeletal pain-grouping AEs, a median time to the first occurrence of these AEs after treatment cessation could not be estimated.

**Table 3:** Clinically notable AE groups (all grades) among patients who continued into the second 48 weeks of the TFR phase (*n* = 100)^a^

Patients, *n* (%)	Consolidation phase (*n* = 100)	TFR phase	
		First 48 weeks (*n* = 100)	Second 48 weeks (*n* = 100)
Cardiovascular events	3 (3.0)	2 (2.0)	1 (1.0)
Ischemic cerebrovascular events	1 (1.0)	1 (1.0)	0
Ischemic heart disease	1 (1.0)	0	1 (1.0)
Peripheral arterial occlusive disease	1 (1.0)	1 (1.0)	0
Musculoskeletal pain	17 (17.0)	34 (34.0)	9 (9.0)
Fluid retention	3 (3.0)	4 (4.0)	4 (4.0)
Edema and other fluid retentions	2 (2.0)	3 (3.0)	4 (4.0)
Severe	1 (1.0)	1 (1.0)	0
Hepatotoxicity	2 (2.0)	2 (2.0)	0
Cardiac failure	0	1 (1.0)	0
Rash	5 (5.0)	1 (1.0)	1 (1.0)
Myelosuppression (thrombocytopenia)	1 (1.0)	0	0
Pancreatitis	1 (1.0)	0	0
Significant bleeding	0	0	1 (1.0)
Gastrointestinal hemorrhage	0	0	1 (1.0)

*AE* adverse event, *TFR* treatment-free remission

^a^Each listed AE group includes a predefined set of individual AEs. Reported frequencies include all patients with ≥ 1 new or worsening AE in the group reported during the indicated study period

## Conclusions

Results of the 96-week analysis affirm findings from the 48-week analysis. The TFR rate was 48.9% at 96 weeks and 51.6% at 48 weeks (Hochhaus et al. 2017a), suggesting a very low risk of relapse in patients remaining in TFR for > 48 weeks. As already noted in the 48-week analysis (Hochhaus et al. 2017a), most TFS events occurred during the first 24 weeks of TFR, after which the Kaplan–Meier curve for TFS approached a plateau. Of the 98 patients in the TFR phase at 48 weeks, only 3 exited this phase due to loss of MMR during the second 48 weeks, and 2 others discontinued the study despite remaining in MMR. Patients reinitiating nilotinib following loss of MMR promptly regained molecular responses; 98.9% regained MMR and 92.0% regained MR^4.5^ by the data cut-off, demonstrating that temporary treatment cessation is safe in patients who experience molecular relapse. This 96-week TFR rate is comparable to TFR rates reported in other studies (Etienne et al. 2017; Ross et al. 2013; Mahon et al. 2010; Nakamae et al. 2017; Mori et al. 2015; Rea et al. 2017; Saussele et al. 2017; Rousselot et al. 2014; Lee et al. 2016; Imagawa et al. 2015), including the ENESTop study (96-week TFR rate of 53.2%) (Mahon et al. 2018), and the high response rate following nilotinib reinitiation is also consistent with results from ENESTop (Mahon et al. 2018).

The frequency of AEs, including musculoskeletal pain AEs, decreased during the second 48 weeks of the TFR phase. This pattern of musculoskeletal pain AEs occurring early during the TFR phase and decreasing later is similar to that reported following second-line nilotinib cessation in the ENESTop study (Mahon et al. 2018). A TKI withdrawal syndrome of musculoskeletal pain has also been reported following imatinib or dasatinib treatment cessation in other TFR studies, although it has not been rigorously characterized, and the underlying biological mechanisms are currently unknown (Lee et al. 2016; Richter et al. 2014; Saußele et al. 2016; Shah et al. 2017).

Importantly, TFR did not negatively affect patients’ clinical outcomes. There were no reports of progression to AP/BC or deaths due to CML, and AE frequencies generally decreased following nilotinib cessation. The number of cardiovascular events was lower in the second year of TFR than in the first year of TFR or during nilotinib consolidation, although much larger numbers of patients would be required to examine whether the vascular risk associated with nilotinib treatment is reversed during TFR. Together with the rapid restoration of DMR in patients who reinitiated treatment and the decreasing frequency of musculoskeletal pain AEs over time during TFR, these results suggest that TFR is safe and does not adversely impact patients’ clinical outcomes or lead to TKI resistance.

We previously performed multivariate logistic regression analyses to evaluate whether patient baseline characteristics (including sex, age, and duration of nilotinib treatment or MR^4.5^ prior to study entry) were predictive of successful TFR (Hochhaus et al. 2017a). However, none of the evaluated characteristics were found to be strong predictors of successful TFR. In this 96-week analysis, the subgroup of patients having low Sokal risk scores at diagnosis and the subgroup maintaining MR^4.5^ consistently throughout the consolidation phase both had numerically higher TFR rates. In agreement with this, the STIM1 study reported that low and intermediate Sokal risk scores were associated with lower molecular recurrence rates (Etienne et al. 2017). However, the Korean Imatinib Discontinuation and STOP Second-Generation TKI studies reported finding no association between Sokal risk scores and TFR rates, highlighting that predictors of successful TFR are not completely defined (Lee et al. 2016; Rea et al. 2017). Other studies have reported that TFR rates may be higher in patients with longer durations of TKI therapy (Etienne et al. 2017; Lee et al. 2016; Saussele et al. 2017) and those with longer DMR duration prior to treatment cessation (Saussele et al. 2017). As more patients attempt TFR, especially with CML treatment guidelines now noting that TFR may be attempted outside of clinical trials (National Comprehensive Cancer Network 2017; Hochhaus et al. 2017b), the identification of predictors of successful TFR will be critical to enabling physicians to determine which patients would be most likely to benefit from treatment cessation.

We additionally found that a deeper molecular response at 12 weeks of TFR appeared to be predictive of maintaining TFR through 96 weeks. Thus, for patients with MMR but not MR^4.5^ at 12 weeks, close monitoring is especially critical, since they may have an increased risk of molecular relapse. This is consistent with the fact that many TFR studies reported that most molecular relapses occurred early in TFR (Etienne et al. 2017; Mahon et al. 2010; Ross et al. 2013; Rousselot et al. 2014; Lee et al. 2016; Mori et al. 2015; Imagawa et al. 2015; Rea et al. 2017; Hughes et al. 2016; Nakamae et al. 2017; Saussele et al. 2017) and highlights the importance of frequent monitoring early during TFR. However, although molecular relapses tend to be less frequent later in TFR, regular monitoring remains essential for all patients during long-term follow-up, because late molecular relapses do occur. Every patient who lost MR^4^ during the first 12 weeks of the TFR phase went on to lose MMR within the first 48 weeks. We have demonstrated that loss of MMR is a safe and reproducible trigger for retreatment with nilotinib; for patients in whom *BCR-ABL1*^IS^ levels rise to above 0.01% within the first 12 weeks, diligent follow-up is critical to ensure that treatment is resumed as soon as loss of MMR is evident.

The updated results from ENESTfreedom presented here demonstrate the durability of TFR following frontline nilotinib and continue to demonstrate the safety of TFR. The results suggest that patients with consistent MR^4.5^ throughout the year prior to nilotinib cessation or low Sokal risk scores at diagnosis have numerically higher TFR rates. Strong predictors of successful TFR after stopping nilotinib remain to be identified. Overall, these findings support the use of frontline nilotinib in patients with newly diagnosed CML-CP for whom TFR might be a future treatment goal.

## References

[CR1] Boquimpani CM, Szczudlo T, Mendelson E, Benjamin K, Masszi T (2014) Attitudes and perceptions of patients (pts) with chronic myeloid leukemia in chronic phase (CML-CP) toward treatment-free remission (TFR). Blood 124(21):[abstract 4547]

[CR2] Etienne G, Guilhot J, Rea D et al (2017) Long-term follow-up of the French Stop Imatinib (STIM1) study in patients with chronic myeloid leukemia. J Clin Oncol 35(3):298–305 28095277 10.1200/JCO.2016.68.2914

[CR3] Hochhaus A, Saglio G, Hughes TP et al (2015) Impact of treatment with frontline nilotinib (NIL) vs imatinib (IM) on sustained deep molecular response (MR) in patients (pts) with newly diagnosed chronic myeloid leukemia in chronic phase (CML-CP). Blood 126(23):[abstract 2781]

[CR4] Hochhaus A, Saglio G, Hughes TP et al (2016) Long-term benefits and risks of frontline nilotinib vs imatinib for chronic myeloid leukemia in chronic phase: 5-year update of the randomized ENESTnd trial. Leukemia 30(5):1044–1054. 10.1038/leu.2016.5 26837842 10.1038/leu.2016.5 PMC4858585

[CR5] Hochhaus A, Masszi T, Giles FJ et al (2017a) Treatment-free remission following frontline nilotinib in patients with chronic myeloid leukemia in chronic phase: results from the ENESTfreedom study. Leukemia 31(7):1525–1531. 10.1038/leu.2017.63 28218239 10.1038/leu.2017.63 PMC5508077

[CR6] Hochhaus A, Saussele S, Rosti G et al (2017b) Chronic myeloid leukemia: EMSO clinical practice guidelines for diagnosis, treatment, and follow-up. Ann Oncol 28(suppl 4):iv41-iv51. 10.1093/annonc/mdx219 28881915 10.1093/annonc/mdx219

[CR7] Hughes TP, Ross DM (2016) Moving treatment-free remission into mainstream clinical practice in CML. Blood 128(1):17–23. 10.1182/blood-2016-01-694265 27013442 10.1182/blood-2016-01-694265

[CR8] Hughes TP, Boquimpani C, Kim D et al (2016) Treatment-free remission (TFR) in patients (pts) with chronic myeloid leukemia in chronic phase (CML-CP) treated with second-line nilotinib (NIL): first results from the ENESTop study. J Clin Oncol 34(suppl):[abstract 7054]

[CR9] Imagawa J, Tanaka H, Okada M et al (2015) Discontinuation of dasatinib in patients with chronic myeloid leukaemia who have maintained deep molecular response for longer than 1 year (DADI trial): a multicentre phase 2 trial. Lancet Haematol 2(12):e528–e535. 10.1016/S2352-3026(15)00196-9 26686407 10.1016/S2352-3026(15)00196-9

[CR10] Jiang Q, Liu ZC, Zhang SX, Gale RP (2016) Young age and high cost are associated with future preference for stopping tyrosine kinase inhibitor therapy in Chinese with chronic myeloid leukemia. J Cancer Res Clin Oncol 142(7):1539–1547. 10.1007/s00432-016-2159-7 27085529 10.1007/s00432-016-2159-7 PMC11819211

[CR11] Lee SE, Choi SY, Song HY et al (2016) Imatinib withdrawal syndrome and longer duration of imatinib have a close association with a lower molecular relapse after treatment discontinuation: the KID study. Haematologica 101(6):717–723. 10.3324/haematol.2015.139899 26888022 10.3324/haematol.2015.139899 PMC5013960

[CR12] Mahon FX, Rea D, Guilhot J et al (2010) Discontinuation of imatinib in patients with chronic myeloid leukaemia who have maintained complete molecular remission for at least 2 years: the prospective, multicentre Stop Imatinib (STIM) trial. Lancet Oncol 11(11):1029–1035. 10.1016/S1470-2045(10)70233-3 20965785 10.1016/S1470-2045(10)70233-3

[CR13] Mahon FX, Boquimpani C, Kim DW et al (2018) Treatment-free remission following second-line nilotinib treatment in patients with chronic myeloid leukemia in chronic phase: results from the single-arm, phase 2, open-label ENESTop study. Ann Intern Med. 10.7326/M17-1094 29459949 10.7326/M17-1094

[CR14] Mori S, Vagge E, le Coutre P et al (2015) Age and dPCR can predict relapse in CML patients who discontinued imatinib: the ISAV study. Am J Hematol 90(10):910–914. 10.1002/ajh.24120 26178642 10.1002/ajh.24120

[CR15] Nakamae H, Imagawa J, Tanaka H et al (2017) Final study results of discontinuation of dasatinib in patients with CML who maintained deep molecular response for longer than one year (DADI trial) after three years of follow-up. Haematologica 102(s2):77 [abstract P263]

[CR16] National Comprehensive Cancer Network (2017) NCCN clinical practice guidelines in oncology: chronic myeloid leukemia. V3.2018 10.6004/jnccn.2017.0116 28687581

[CR17] Novartis Pharmaceuticals Corporation (2017a) Tasigna (nilotinib) [package insert]. East Hanover, New Jersey

[CR18] Novartis Pharmaceuticals Corporation (2017b) Tasigna (nilotinib) [summary of product characteristics]. Camberley, UK

[CR19] Rea D, Nicolini FE, Tulliez M et al (2017) Discontinuation of dasatinib or nilotinib in chronic myeloid leukemia: interim analysis of the STOP 2G-TKI study. Blood 129(7):846–854. 10.1182/blood-2016-09-742205 27932374 10.1182/blood-2016-09-742205

[CR20] Richter J, Söderlund S, Lübking A et al (2014) Musculoskeletal pain in patients with chronic myeloid leukemia after discontinuation of imatinib: a tyrosine kinase inhibitor withdrawal syndrome? J Clin Oncol 32(25):2821–2823. 10.1200/JCO.2014.55.6910 25071107 10.1200/JCO.2014.55.6910

[CR21] Ross DM, Branford S, Seymour JF et al (2013) Safety and efficacy of imatinib cessation for CML patients with stable undetectable minimal residual disease: results from the TWISTER study. Blood 122(4):515–522. 10.1182/blood-2013-02-483750 23704092 10.1182/blood-2013-02-483750

[CR22] Rousselot P, Charbonnier A, Cony-Makhoul P et al (2014) Loss of major molecular response as a trigger for restarting tyrosine kinase inhibitor therapy in patients with chronic-phase chronic myelogenous leukemia who have stopped imatinib after durable undetectable disease. J Clin Oncol 32(5):424–430. 10.1200/JCO.2012.48.5797 24323036 10.1200/JCO.2012.48.5797

[CR23] Saußele S, Richter J, Hochhaus A, Mahon FX (2016) The concept of treatment-free remission in chronic myeloid leukemia. Leukemia 30(8):1638–1647. 10.1038/leu.2016.115 27133824 10.1038/leu.2016.115 PMC4980559

[CR24] Saussele S, Richter J, Guilhot J et al (2017) “Duration of deep molecular response” has most impact on the success of cessation of tyrosine kinase inhibitor treatment in chronic myeloid leukemia—results from the EURO-SKI trial. Blood 130(suppl 1):[abstract 313]

[CR25] Shah NP, Gutiérrez VG, Jiménez-Velasco A et al (2017) Dasatinib discontinuation in patients (pts) with chronic-phase chronic myeloid leukemia (CML-CP) and stable deep molecular response (DASFREE). Blood 130(Suppl 1):[abstract 314] 10.1080/10428194.2019.1675879 31647335

[CR26] Villemagne Sanchez LA, O’Callaghan C, Gough K et al (2018) Patient perceptions of treatment-free remission in chronic myeloid leukemia. Leuk Lymphoma 59(2):406–415. 10.1080/10428194.2017.1337114 28617066 10.1080/10428194.2017.1337114

